# Entomopathogenic fungi, plasma-activated water, and their synergistic effects for sustainable management of *Lipaphis erysimi*

**DOI:** 10.3389/ffunb.2026.1834224

**Published:** 2026-06-30

**Authors:** Alviti Kankanamalage Hasith Priyashantha, Sarayut Pittarate, Thanandon Siripan, Jutamart Monkai, Saowaluck Tibpromma, Nuttapol Noirungsee, Norrapon Vichiansan, Samantha C. Karunarathna, Patcharin Krutmuang, Saisamorn Lumyong

**Affiliations:** 1 Department of Biology, Faculty of Science, Chiang Mai University, Chiang Mai, Thailand; 2 Office of Research Administration, Chiang Mai University, Chiang Mai, Thailand; 3 Department of Entomology and Plant Pathology, Faculty of Agriculture, Chiang Mai University, Chiang Mai, Thailand; 4 Center for Yunnan Plateau Biological Resources Protection and Utilization and Yunnan International Joint Laboratory of Fungal Sustainable Utilization in South and Southeast Asia, College of Biology and Food Engineering, Qujing Normal University, Qujing, China; 5 Center of Excellence in Microbial Diversity and Sustainable Utilization, Faculty of Science, Chiang Mai University, Chiang Mai, Thailand; 6 Multidisciplinary Center, Faculty of Engineering, Chiang Mai University, Chiang Mai, Thailand; 7 Center of Omics for High-Value Agriculture (AgOmics-CMU), Chiang Mai University, Chiang Mai, Thailand; 8 Academy of Science, The Royal Society of Thailand, Bangkok, Thailand

**Keywords:** conidial germination, *Cordyceps javanica*, entomopathogenic fungi, insect mortality, mustard aphid, plasma treatment, reactive oxygen and nitrogen species

## Abstract

Entomopathogenic fungi (EPF) are widely recognized as effective and environmentally sustainable biological control agents for a broad spectrum of agricultural insect pests. The increasing complexity and intensification of contemporary agricultural systems require the identification and development of more virulent and resilient fungal strains to ensure consistent pest suppression. In this study, ten EPF isolates representing *Beauveria bassiana, Cordyceps javanica*, and *Metarhizium anisopliae* were selected based on preliminary laboratory screens, and the insecticidal effectiveness of these isolates was tested against the mustard aphid, *Lipaphis erysimi*, along with the direct and interactive effects of plasma-activated water (PAW). Here, the PAW was generated using a surface dielectric barrier discharge (sDBD) plasma system, producing highly acidic water enriched with reactive oxygen and nitrogen species. Direct and daily application of PAW at concentrations of 50, 100, and 1,000 ppm resulted in complete aphid mortality within seven days at all tested concentrations. Conidial germination assays showed that lower PAW concentrations had less effect, whereas higher concentrations significantly inhibited germination. In certain isolates, the combined application of 100 ppm PAW and EPF produced greater aphid control than single EPF treatments. Overall, a single application of EPF achieved 87.67% aphid control, whereas the combined treatment achieved 99.67% mortality. Based on Lethal Time (LT_50_) values, *C. javanica* SLLC-Cj 24 (single application average 6.93 days; combined application average 6.60 days) was the most virulent strain, followed by SLLC-Cj 11 (single application average 7.17 days; combined application average 6.65 days). Among the tested fungal strains, *B. bassiana* SLLC-Bb 12 demonstrated the strongest synergistic interaction with PAW, followed by *C. javanica* SLLC-Cj 1. These findings demonstrate that PAW possesses intrinsic insecticidal activity and can enhance overall mortality when integrated with EPF. However, isolate- or strain-specific interactions may influence mortality and infection rates, highlighting the need to select fungal cultures, optimize concentration, and exposure parameters for synergistic biocontrol strategies.

## 1 Introduction

Entomopathogenic fungi (EPF) are the naturally occurring organisms that infect a wide range of insect groups. Infection by EPF primarily occurs through direct penetration of the host insect cuticle, followed by proliferation within the host hemocoel, nutrient acquisition, emergence from the cadaver, and subsequent conidiation (Ying, 2024). During infection, insects may die within a few days to several weeks, depending on the virulence of the pathogenic fungus. The dispersal conidia serve as the infective inoculum for subsequent disease cycles when they encounter suitable hosts. Thus, conidia play a crucial role as the primary propagules responsible for infection and transmission in EPF (Priyashantha et al., 2025).

Increasing evidence highlighting the adverse effects of synthetic pesticides, including biodiversity loss, soil degradation, and risks to human health, has led to a decline in their use (Tang et al., 2021; Okagu et al., 2023; Zhou et al., 2025). The recent development of green agriculture has led to the search for alternative, effective agricultural practices that replace historical practices of synthetic fertilizer and pesticide inputs (Zou et al., 2023; Vivekanandhan et al., 2021; Shaizee et al., 2026). Insects pose a significant impact on agricultural commodities; estimates indicate that about 10.8% of worldwide crop losses are due to insect pests. The recent decline in agricultural production showed 18–26%, and economic losses valued at $470 billion (Deka et al., 2021). Considering those, it is now evident that controlling insect pests through eco-friendly approaches is urgently needed. Among these, myco-biocontrol represents an environmentally sound, cost-effective, and sustainable strategy that utilizes EPF as natural enemies for pest management (Jaiswal et al., 2022; Sandhu et al., 2012). Afore-described effective behavior of EPF, utilized today in the commercial agriculture sector to control insect pests in many countries in the world. Currently, there are about 171 commercial EPF products available, of which 67.8% consist of *Beauveria bassiana* and *Metarhizium anisopliae* (shared in almost equal proportions), 5.8% *Isaria fumosorosea*, and 4.1% *Beauveria brongniartii* (Jaronski, 2023). Recent studies have demonstrated the application of EPF in targeting several major agricultural pests, including species belonging to the order Blattodea (Ambele et al., 2020), Coleoptera (Keyser et al., 2014), Hemiptera (Clifton et al., 2018), and Lepidoptera (Silva et al., 2020) in cash crops such as wheat (*Triticum aestivum*), cocoa (*Theobroma cacao*), soybean (*Glycine max*), and tomato (*Solanum lycopersicum*), respectively.

With advances in EPF-based biocontrol research, efforts have also been made to enhance their effectiveness through integrated approaches. Several studies have reported promising results when EPF were combined with botanicals (Fernández-Grandon et al., 2020), synthetic pesticides (Zuparov and Ablazova, 2024), and natural predators (Alkhaibari et al., 2018). Plasma-activated water (PAW) is one of the novel cutting-edge technologies that has received considerable attention from researchers over the last decade due to its non-thermal and non-toxic mode of action (Royintarat et al., 2020; Antoni et al., 2025; Long et al., 2026). The technique was developed about a decade ago and is particularly recognized as a potential disinfectant for food and medical/healthcare instruments (Ma et al., 2015; Zhao et al., 2020).

Plasma-activated water is produced by treating water with cold atmospheric plasma under controlled conditions, including plasma-forming voltage, carrier gas, temperature, pulse duration, and frequency (Soni et al., 2021). Cold plasma, or non-thermal plasma, is usually created by applying thermal discharge, such as electromagnetic, electric, microwave, or radio-frequency fields, to gases in air, including Argon (Ar), Helium (He), Nitrogen (N_2_), or Oxygen (O_2_) at low or atmospheric pressure. When plasma interacts with water, various reactive oxygen and nitrogen species (RONS) are generated, which are widely recognized as key contributors to the biological efficacy of PAW (Moszczyńska et al., 2023; Kim and Kim, 2021; Jangra et al., 2024). Those RONS include short-lived species such as hydroxyl radicals (OH), nitric oxide (NO), and peroxynitrite (ONOO^-^), which typically have half-lives ranging from nanoseconds to several seconds and rapidly react to form more stable compounds. In contrast, longer-lived species such as hydrogen peroxide (H_2_O_2_), ozone (O_3_), nitrite (NO_2_^-^), and nitrate (NO_3_^-^) persist for minutes to days, prolonging the system’s reactivity (Ahmed et al., 2017; Wong et al., 2023).

As a result, PAW is characterized by lower pH (acidic conditions), higher oxidation–reduction potential, increased electrical conductivity, and higher salinity relative to untreated water. These physicochemical changes underpin their strong antimicrobial and bioactive properties (Wong et al., 2023). Rather than considering washing disinfectants, today it has also shown the importance of anti-cancer therapy, anti-inflammatory agent that accelerates wound healing, tooth bleaching, treatment of oral infectious diseases, control of industrial pollutants (e.g., improving the wettability of coal dust), and so on (Milhan et al., 2022; Zheng et al., 2025). Despite this, the promising results of PAW in controlling insect pests have opened new avenues for research; however, these aspects remain poorly investigated to date (Ten Bosch et al., 2017).

Under these circumstances, the primary objective of this study was to evaluate the biocontrol potential of newly isolated, naturally occurring EPF strains against the mustard aphid, *Lipaphis erysimi*, in combination with PAW. Specifically, the study aimed to: (i) determine the independent efficacy of PAW in controlling mustard aphid; (ii) investigate the effects of PAW exposure on EPF conidial viability; and (iii) assess the potential synergistic interaction between EPF and PAW treatments in enhancing aphid mortality. By integrating microbial biocontrol agents with a novel oxidative treatment, this research seeks to provide insights into the feasibility of combined strategies for sustainable aphid management.

## 2 Methodology

### 2.1 Source of EPF

Entomopathogenic fungi used in this study were obtained from the Saisamorn Lumyong Laboratory Culture Collection (SLLC), Faculty of Science, Chiang Mai University, and the Insect Pathology Laboratory (IPL), Division of Entomology, Department of Entomology and Plant Pathology, Faculty of Agriculture, Chiang Mai University. The fungal isolates were derived from our previous field surveys conducted in Northern Thailand (Priyashantha et al., 2024). During these surveys, numerous EPF isolates were recovered from soil and insect cadavers collected in areas associated with the Doi Suthep–Pui National Park Mountain Forest ecosystem and other natural habitats (**Supplementary Table 1**).

### 2.2 Selection of fungal isolates for the present study

Of the 68 fungal strains, the 10 most virulent isolates were selected for further evaluation based on assays conducted using *Tenebrio molitor* as a potential model host (Canteri de Souza et al., 2018; Chang et al., 2021). Fungal virulence was assessed using the contact–whole plate method. Each isolate was cultured on 90-mm PDA plates (three replicates) for 14 days (Beemrote et al., 2024). Ten healthy fourth–sixth instar *T. molitor* larvae were then introduced onto each fungal culture after 24 h of starvation to minimize avoidance behavior. The plates were partially sealed and incubated at 28 ± 2 °C in the dark, with a relative humidity of 60–70%, for 10 days. A commercial *M. anisopliae* product (PP-Metha) was used as a positive control, while larvae placed on less-moist PDA plates served as the negative control. Larval mortality was recorded daily and verified by mechanical stimulation (Meier et al., 2023), mycosis, or detection of internal mycelial growth. The selected fungal strains belonged to three species groups: *B. bassiana* (BbIPLCMU-1, BbIPLCMU-2, BbIPLCMU-18, SLLC-Bb 12), *Cordyceps javanica* (SLLC-Cj 1, SLLC-Cj 2, SLLC-Cj 11, SLLC-Cj 24), and *M. anisopliae* (MaIPLCMU-5, MaIPLCMU-10).

### 2.3 Host plant cultivation, aphid rearing, and maintenance

Chinese kale (*Brassica oleracea*) plants were grown from commercially sourced seeds obtained from Chia Tai Company, Bangkok, Thailand. Seeds were sown in plastic pots (5.08 cm diameter), and the seedlings were maintained under insect-proof netting to prevent pest attacks. Mustard aphids (*L. erysimi*) were collected from the Mae Hia Agricultural Demonstration and Training Center, Division of Entomology, Department of Entomology and Plant Pathology, Faculty of Agriculture, Chiang Mai University, and subsequently established and maintained as a laboratory population at the IPL. To maintain a healthy population, aphids were continuously raised on Chinese kale plants and transferred to freshly potted plants every week. When host plants reached the 4–6 leaf stage, aphid nymphs were carefully transferred onto the leaves using a fine paintbrush moistened with sterile distilled water to minimize handling injury. Infested plants were then placed in a greenhouse under controlled environmental conditions (25 ± 1 °C, 60 ± 5% relative humidity, and a 16:8 h light: dark photoperiod) to facilitate aphid multiplication, following previously described procedures (Bayındır Erol et al., 2020). Further, all experimental units were maintained under insect-proof netting.

### 2.4 Preparation of PAW

**Figure 1:**
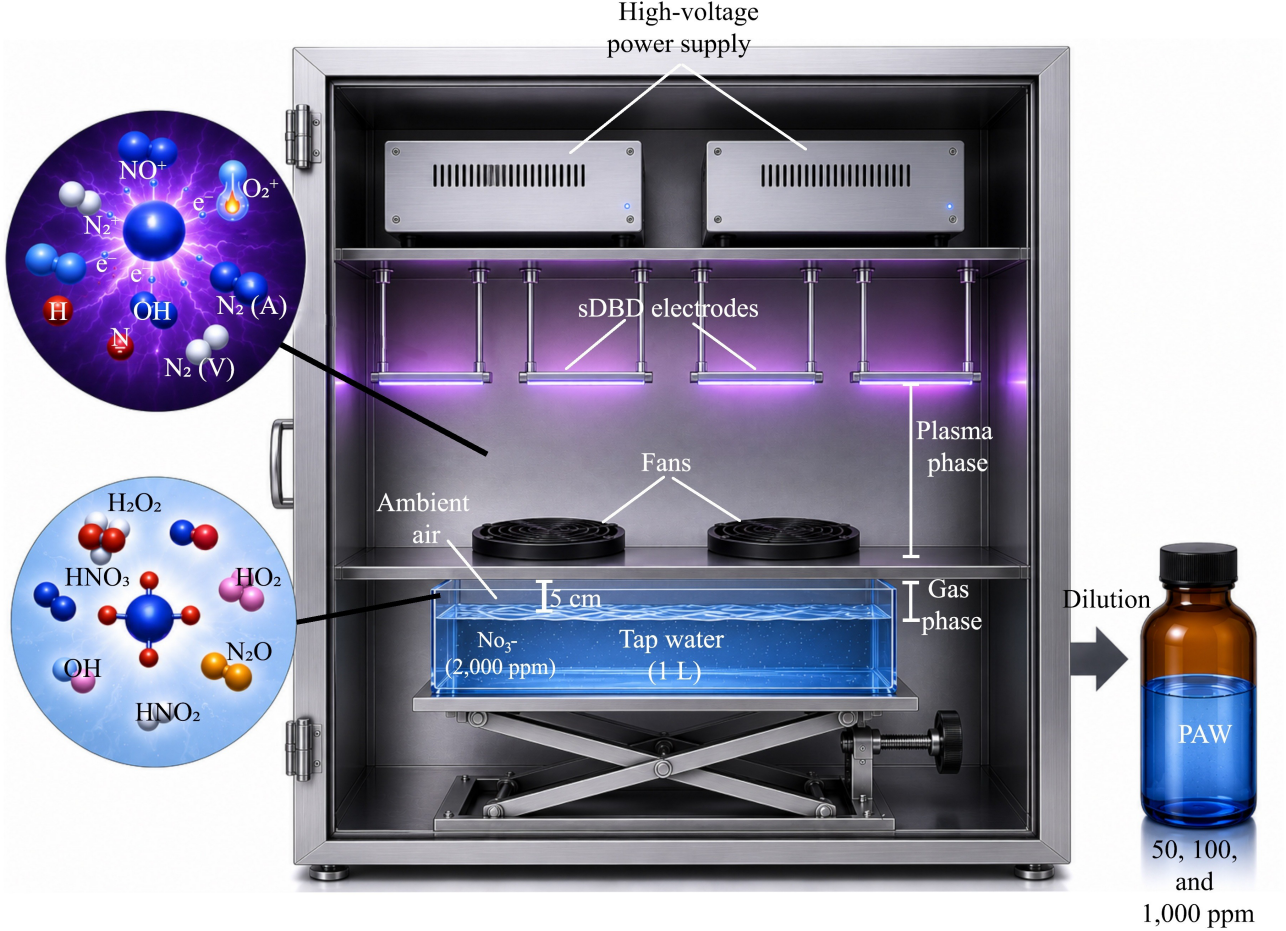
Schematic experimental setup for the generation of PAW using an sDBD plasma system. The PAW was further diluted to 50, 100, and 1,000 ppm for use.

Plasma-activated water was prepared by exposing 1,000 mL of tap water to ambient air plasma generated using a surface dielectric barrier discharge (sDBD) system, as shown in **Figure 1**.

The plasma source was powered by two commercial ozone high-voltage transformers (AC 220 V, 50 Hz; output voltage 3.5–5.0 kV; average total power consumption 220 W). Four planar dielectric-covered electrodes were arranged above the water surface to ensure uniform plasma exposure, with a fixed electrode–water distance of 8 cm. Plasma treatment was conducted without external gas flow. Tap water was exposed to plasma for 150 min to obtain RNS-dominant plasma-activated water. Under these conditions, nitrate (NO_3_^-^) was the dominant long-lived species, reaching an average concentration of approximately 1,889.22 ppm. Although plasma diagnostics such as optical emission spectroscopy (OES) were not included in this study, the reproducibility of PAW chemistry was therefore confirmed *via* stable NO_3_^-^ concentrations, serving as a long-lived chemical marker of plasma–liquid interaction, as measured by ion chromatography (TOSOH IC-8100, Tosoh Bioscience, Japan). The PAW stock solution was subsequently diluted to 50, 100, and 1,000 ppm for aphid and fungal treatments. The detailed operational parameters of the plasma system are summarized in **Supplementary Table 2**.

### 2.5 Effectiveness of PAW on aphids with repeated application

The efficacy of PAW against mustard aphids was evaluated following an adapted protocol based on the methodology described by Ten Bosch et al. (2017). Leaf discs (6 cm in diameter) were excised from fresh, healthy greenhouse-grown Chinese kale and placed individually in sterile Petri dishes containing water agar supplemented with chloramphenicol (10 mg L^-^¹) to minimize bacterial contamination. Each treatment consisted of three replicates, with one leaf disc per dish. The sample size was selected based on comparable EPF and aphid bioassays reported in the literature, where similar replication levels were sufficient to detect treatment effects (Xu et al., 2026). Two adult aphids were introduced onto each leaf disc and allowed to reproduce for 24 h under controlled laboratory conditions. After this period, the adults and excess offspring were carefully removed, leaving exactly ten first-instar nymphs per dish. The nymphs were subsequently treated with PAW at concentrations of 50, 100, and 1,000 ppm once every 24 h for seven consecutive days. Treatments were applied by placing a single 2 µL droplet of PAW directly onto the dorsal surface of each nymph using a calibrated micropipette. Control groups received an equivalent volume (2 µL) of sterile distilled water instead of PAW.

### 2.6 Effect of PAW on fungal conidial germination

The effect of PAW on conidial germination of EPF was evaluated using an *in vitro* germination assay. Three mycelial plugs (10 mm diameter) were excised from the actively growing margins of 14-day-old cultures of each fungal isolate using a sterile cork borer. The plugs were transferred into sterile 1.5 mL microcentrifuge tubes containing 0.75 mL of PAW at concentrations of 50, 100, or 1,000 ppm. The tubes were briefly vortexed to facilitate the release of conidia into suspension, then incubated at 28 ± 2 °C for exposure periods of 0, 30, 60, 90, or 120 min. Each isolate–concentration–exposure time combination was conducted with three independent replicates. At the end of each exposure period, a 3 µL aliquot of the conidial suspension was collected, and three 1 µL droplets were aseptically placed onto PDA plates, evenly spaced to avoid overlap. This procedure was repeated for each replicate suspension. The inoculated plates were incubated at 28 ± 2 °C for 24 h to allow germination. Conidial germination was then assessed under a light microscope at 400× magnification by counting approximately 100 conidia per plate. A conidium was considered germinated when the germ tube length was greater than or equal to the conidium length. The germination percentage was calculated for each treatment.

### 2.7 Application of EPF, PAW, and their combination on *L. erysimi* mortality assessment

Conidia of each EPF isolate were harvested from the surface of two-week-old cultures using the above-mentioned method and suspended in a sterile 0.03% Tween-80 solution. Conidial concentrations were determined using a hemocytometer and adjusted to a final concentration of 1 × 10^7^ conidia/mL for all isolates following established procedures (Kiliç et al., 2018). Fresh leaf discs of Chinese kale were immersed in the respective fungal conidial suspensions (with 0.03% Tween-80 solution) for 15–20 seconds and placed individually into 2-oz plastic containers. The leaf discs were also dipped in plasma-activated water (PAW; 100 ppm) and kept in 2-oz plastic containers. For combined treatments, leaf discs were similarly dipped in fungal conidial suspensions in plasma-activated water (PAW; 100 ppm) before exposure to insects. Each treatment consisted of three replicates, with ten first-instar aphid nymphs introduced into each container. A 0.03% Tween-80 solution was used as the negative control. In total, 66 experimental units were established. Insect mortality (a total of 660 insects) was recorded daily for 12 days.

Mortality was determined based on lack of response to gentle physical stimulation, immobility, and subsequent confirmation of fungal infection through the observation of external mycosis under a stereomicroscope. The median lethal time (LT_50_) was estimated for each fungal isolate under both single (EPF alone) and combined (EPF + PAW) treatments using daily cumulative mortality data.

### 2.8 Data analysis

All statistical analyses were performed using the Statistical Package for the Social Sciences (SPSS) version 23.0 (IBM Corp., Armonk, NY, USA). Conidial germination data were analyzed using repeated-measures analysis of variance (ANOVA) within the general linear model (GLM) framework. Plasma-activated water concentration and fungal isolate were treated as fixed between-subject factors, and exposure time was included as the repeated (within-subject) factor to account for temporal dependence in germination measurements from the same experimental units. When significant interactions were identified, a separate two-way ANOVA (PAW concentration × fungal isolate) was conducted at each time point to clarify treatment effects. Mean separations were determined using Tukey’s honestly significant difference (HSD) test at *p* ≤ 0.05. Percentage data were arcsine square-root transformed prior to analysis to meet assumptions of normality and homogeneity of variance; however, untransformed means are reported for clarity. A repeated-measures general linear model was also used to evaluate temporal effects on conidial germination across isolates. For bioassay experiments assessing aphid mortality, one-way ANOVA was used to compare treatment effects within a single factor (e.g., PAW concentration), and two-way ANOVA was applied to assess the combined effects of EPF and PAW (EPF treatment × PAW application) (Gourgouta and Athanassiou, 2023; Barta et al., 2026).

## 3 Results

### 3.1 Chemical characteristics of PAW

**Figure 2:**
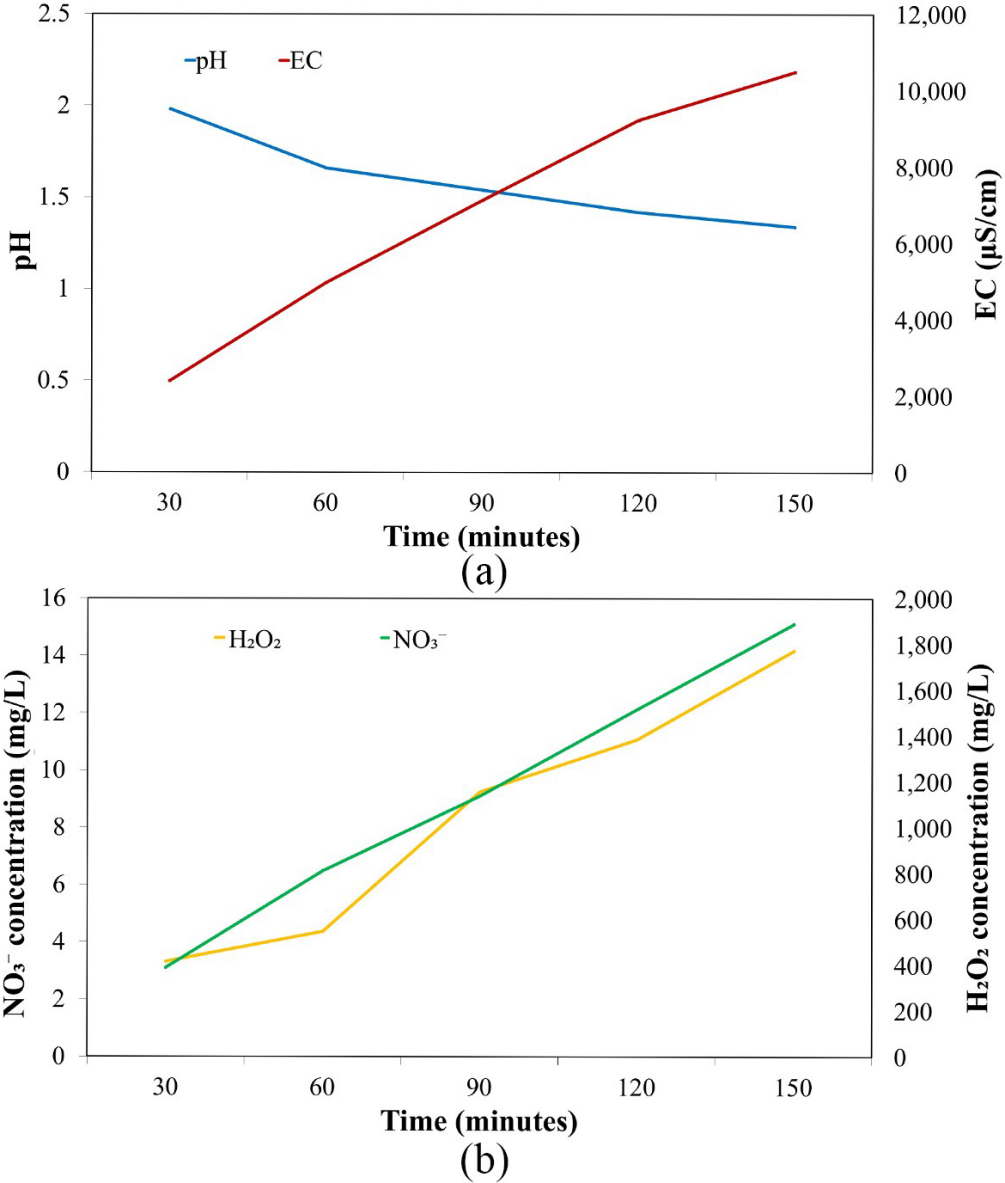
Chemical characteristics of PAW showing the changes in **(a)** pH and electrical conductivity (EC) and **(b)** NO_3_^-^ and H_2_O_2_ concentrations in relation to sDBD treatment time and treated liquid volume.

The physicochemical properties of PAW changed markedly as the sDBD treatment duration increased (30–150 min). A progressive decrease in pH was observed, from 1.98 at 30 min to 1.34 at 150 min, indicating strong acidification of the treated water with prolonged plasma exposure. In parallel, electrical conductivity (EC) increased substantially from 2,389 to 10,491µS/cm, reflecting the continuous accumulation of charged species and dissolved ionic products generated during plasma–liquid interactions (**Figure 2a**). Nitrate (NO_3_^-^) concentration showed a pronounced time-dependent increase, rising from 388.61 to 1,889.22 mg/L, suggesting enhanced formation and dissolution of reactive nitrogen species as plasma treatment time increased. Similarly, H_2_O_2_ concentration increased steadily from 3.32 to 14.18 mg/L, indicating sustained production of long-lived reactive oxygen species (ROS), during sDBD activation (**Figure 2b**). Collectively, these results demonstrate that longer plasma treatment durations intensify the chemical activation of water, resulting in highly acidic PAW with elevated concentrations of reactive oxygen and nitrogen species.

### 3.2 Effectiveness of PAW on *L. erysimi*

**Figure 3:**
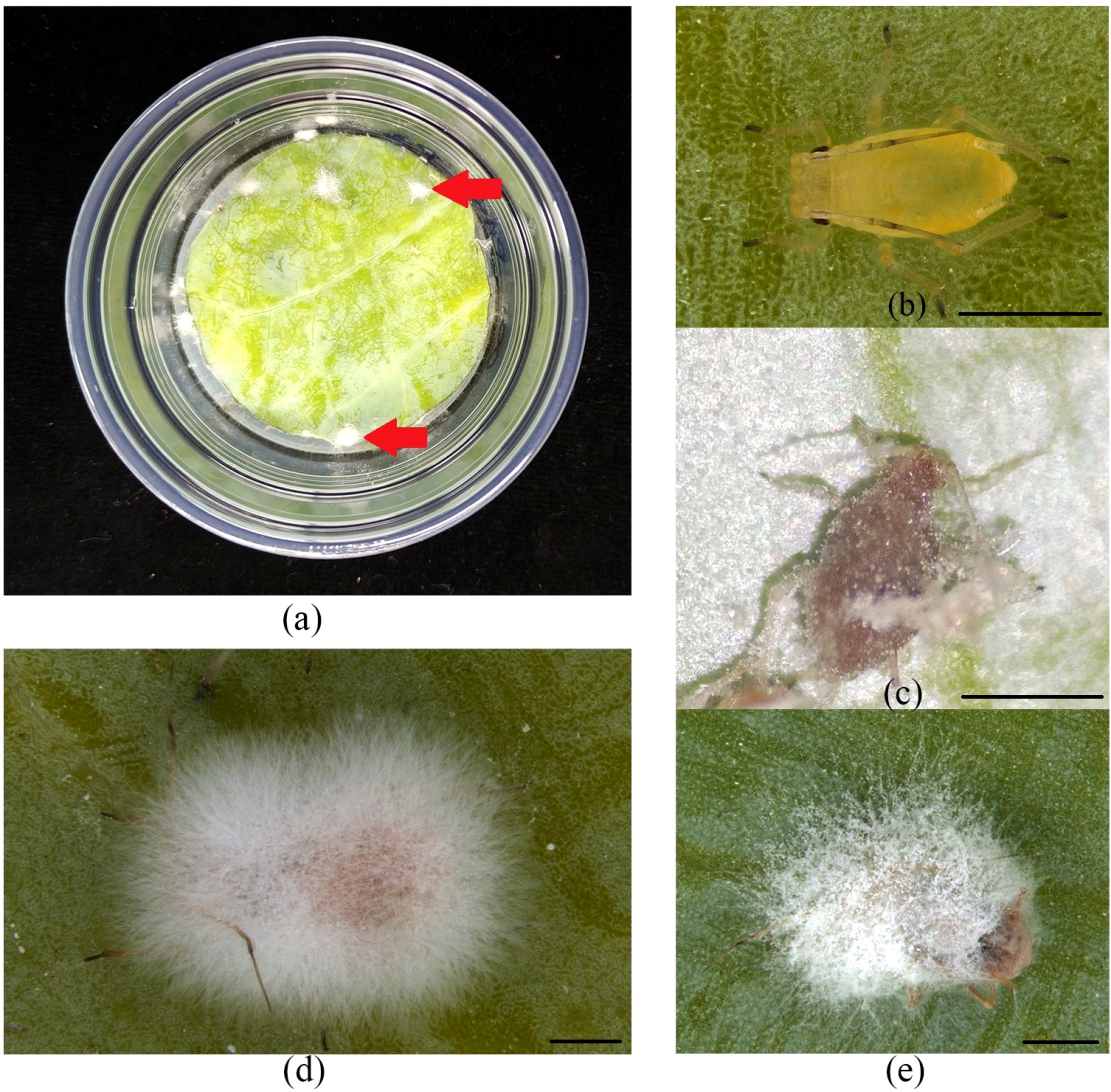
*In vitro* experimental results of aphid control. **(a)** Mycosis shows in aphids (see the arrows) in the experimental setup 12 days after treatment with EPF. **(b)** Healthy aphid nymph (control). **(c)** Dead insects three days after PAW treatment (repeated application), showing abnormal body shape and discoloration **(d)**. The mycelia of *C. javanica* SLLC-Cj 11 covered with the insect cadavers, and **(e)** *B. bassiana* SLLC-Bb 12 showed high sporulation on the 12^th^ day after combining application with PAW. Scale bar: 0.5 mm.

Plasma-activated water caused significant mortality in the insect pest *L. erysimi* with repeated daily application; however, the effect depended on both concentration and exposure duration. Under continuous treatment, all insects died within seven days, regardless of the concentration. However, PAW does not cause immediate mortality in the aphid; instead, it induces cumulative physiological stress that leads to lethality. For example, some treated insects have shown an unhealthy body color (brown) and a swollen abdomen (**Figure 3c**). By day three, PAW treatments differed significantly, with mortality increasing with concentration and reaching a maximum at 1,000 ppm (33.33 ± 5.77%), while no mortality occurred in the control. Note that no sudden death of the insects was noticed, as in the first two days of the experiment.

By day five, cumulative mortality increased sharply in all PAW treatments, with the 1,000 ppm treatment producing the highest mortality (76.67 ± 5.77%), followed by 100 ppm (60.00 ± 10.00%) and 50 ppm (53.33 ± 5.77%). In contrast, mortality in the distilled water control remained negligible (3.33 ± 5.77%), and no mortality was recorded in the untreated control. Treatment effects were highly significant at this time point (*p* < 0.05).

**Figure 4:**
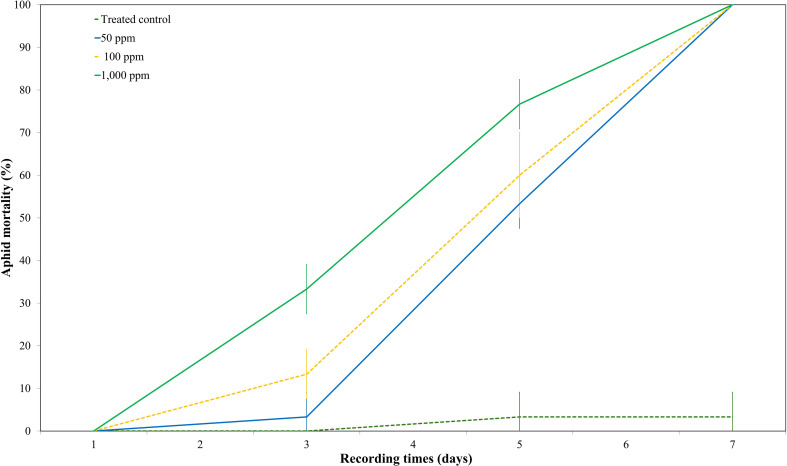
Time-dependent mortality of aphids treated with plasma-activated water (PAW). First-instar aphids were treated daily with PAW at 50, 100, and 1,000 ppm. Values represent mean ± SEM (n = 3). Water-treated aphids served as the control. Statistical analysis was performed only on days three and five because there was no variance in days one and seven.

By day seven, all PAW concentrations resulted in complete (100%) aphid mortality and differed significantly from both control groups, which remained below 3% mortality. The strong treatment effect on day seven (*p* < 0.05) indicates a robust insecticidal response to repeated PAW exposure (**Figure 4**).

### 3.3 Effect of PAW on fungal spores/conidial germination assay

The three-way ANOVA revealed that conidial germination was significantly influenced by fungal isolate and PAW concentration, while exposure time had no significant main effect. Specifically, isolate had a highly significant effect (F_9,300_ = 163.19, *p* < 0.001), indicating substantial variability in germination among the tested isolates. PAW concentration also significantly affected germination (F_2,300_ = 48.36, *p* < 0.001), whereas the effect of exposure time was not statistically significant (F_4,300_ = 2.33, *p* = 0.056). Among interaction terms, only the isolate × concentration interaction was significant (F_18,300_ = 17.46, *p* < 0.001), suggesting that the response of isolates to PAW varied depending on concentration.

**Figure 5:**
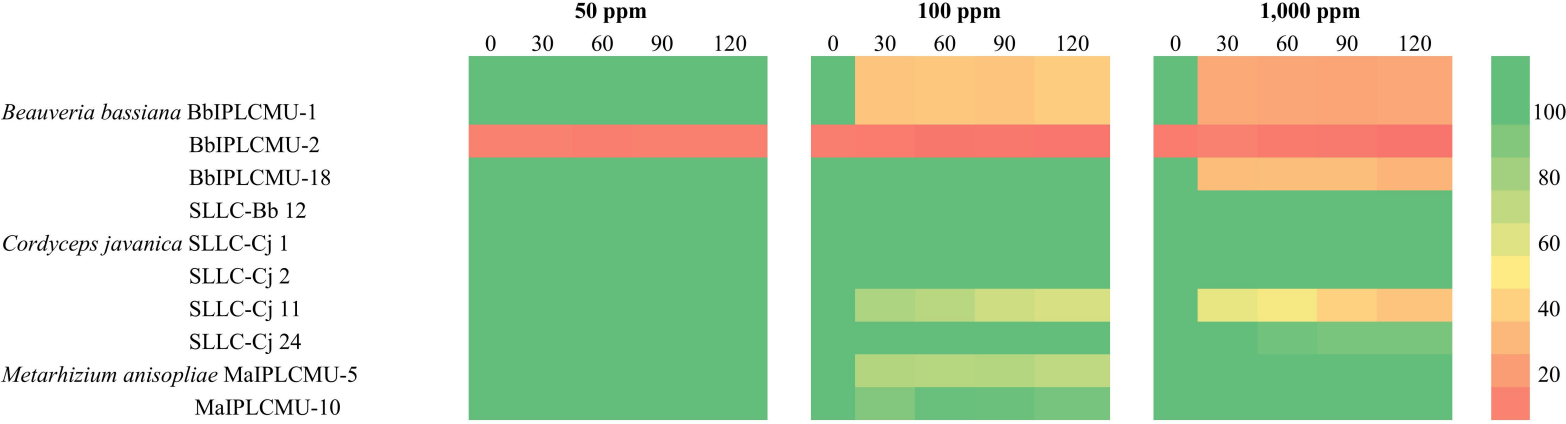
Time-dependent response (%) of conidial germination of fungal isolates to three PAW concentrations. Heatmap showing the mean responses of ten entomopathogenic fungal isolates (*B. bassiana, C. javanica*, and *M. anisopliae*) measured at 0, 30, 60, 90, and 120 min under 50, 100, and 1,000 ppm. Color intensity represents the mean response (%) on a 0–100 scale (see color bar).

At 50 ppm PAW, *B. bassiana* BbIPLCMU-2, and *C. javanica* SLLC-Cj 11 showed reduced germination. In contrast, all other isolates showed consistently high germination (100%) and did not differ significantly among themselves. At 100 ppm PAW, several isolates, including *B. bassiana* BbIPLCMU-2 and BbIPLCMU-1, *M. anisopliae* MaIPLCMU-5, and *C. javanica* SLLC-Cj 11, showed a significant reduction in spore germination. Exposure to PAW at 1,000 ppm resulted in pronounced inhibition of conidial germination in isolates BbIPLCMU-1, BbIPLCMU-18, BbIPLCMU-2, and SLLC-Cj 11, whereas several other isolates remained unaffected across all incubation periods (**Figure 5**). For concentration effects, germination at 50 ppm was significantly higher (90.9% of overall germination) than at 100 ppm (80.59%) and 1,000 ppm (74.53%), whereas no significant difference was observed between 100 ppm and 1,000 ppm. Exposure time did not show significant pairwise differences, with all time points forming a single homogeneous group.

### 3.4 Application of EPF, PAW, and a combination of both on *L. erysimi* mortality

The application of EPF alone and the combination with PAW demonstrated strong insect-killing ability with visible mycosis in insect cadavers (**Figure 3a**). Unlike repeated experimental results, the one-time application (leaf disc dipping method) of PAW didn’t cause any effects on insects, and throughout the experiment period, no deaths of insects were reported, as shown in the positive treatment. Furthermore, no apparent morphological differences were observed between treated and healthy insects (**Figure 3b**). However, the combined approach of EPF and PAW is interesting in enhancing the insect mortality in particular fungal groups (**Figures 3d, e**), showing great fungal infection, also with higher sporulation.

**Figure 6:**
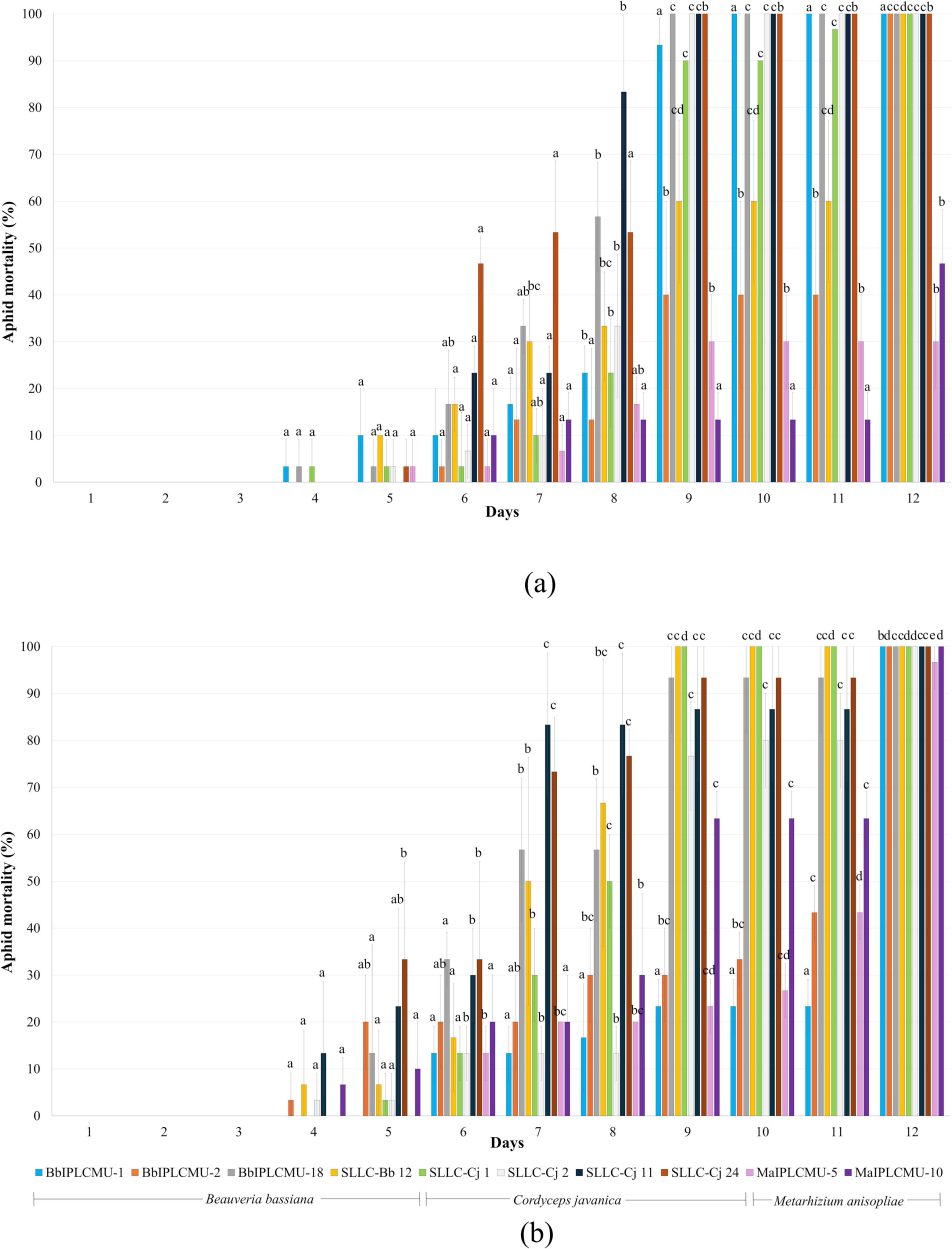
Effect of **(a)** single application of EPF and **(b)** combined with PAW on aphid mortality (%). Different letters above bars indicate statistically significant differences among treatments, based on the pairwise comparisons among strains within each day (one-way ANOVA, *p* < 0.05).

According to Tukey’s HSD *post hoc* analysis, most isolates exhibit significant mortality by the ninth day after treatment in both experiments. Overall, compared with a single application of EPF, the combined EPF–PAW treatment provided better aphid control. A two-way ANOVA revealed that on the 12th day of treatment, EPF combined with PAW had a significant effect on insect mortality. In the single application, by day 12, isolates MaIPLCMU-5 and MaIPLCMU-10 show significantly lower mortality than the others, while the rest show 100% mortality (**Figure 6a**). However, in the combined application, except for MaIPLCMU-5 (96.7%), all other isolates show complete mortality at the end of the experiment (**Figure 6b**). The combined application showed significantly higher mortality (99.67%) than EPF alone (87.67%). This indicates a strong and biologically meaningful effect of PAW on aphid mortality. Another consideration is the effect of the fungal strain. Statistical evidence indicated that the mortality of insects is significantly dependent on the fungal strain, demonstrating substantial variability in aphid mortality among fungal isolates. Notably, a significant interaction of PAW and fungal strain was observed, indicating that the effect of PAW on aphid mortality differed among isolates.

According to the probit analysis, under EPF-only application, SLLC-Cj 24 exhibited the fastest insecticidal activity, with an LT_50_ of 6.93 days (95% CI: 6.62–7.25 days; χ² = 27.86, df = 34, *p* = 0.762). This was followed by SLLC-Cj 11, with an LT_50_ of 7.17 days (95% CI: 6.90–7.44 days; χ² = 17.48, df = 34, *p* = 0.991), and BbIPLCMU-18, which showed an LT_50_ of 7.31 days (95% CI: 7.01–7.63 days; χ² = 33.76, df = 34, *p* = 0.479). In contrast, MaIPLCMU-5 and MaIPLCMU-10 exhibited substantially slower mortality responses, with LT_50_ estimates approaching the upper limit of the observation period, indicating relatively slow mortality progression and reduced virulence. In the combined application, SLLC-Cj 24 exhibited the highest virulence with the lowest LT_50_ value of 6.60 days (χ² = 60.953, df = 34, p = 0.003), followed closely by SLLC-Cj 11 with an LT_50_ of 6.65 days (χ² = 69.21, df = 34, *p* = 0.001). SLLC-Bb 12 showed a comparatively higher LT_50_ of 6.99 days (χ² = 48.839, df = 34, *p* = 0.048). In contrast, BbIPLCMU-1 recorded a substantially higher LT_50_ of 10.79 days (χ² = 41.72, df = 34, *p* = 0.170), indicating the lowest virulence among the tested isolates. Moreover, among the tested isolates, *B. bassiana* SLLC-Bb 12 exhibited the strongest synergistic interaction with PAW, showing the greatest reduction in LT_50_ (−1.91 days), followed by *C. javanica* SLLC-Cj 1 (−0.77 days). In contrast, BbIPLCMU-1 demonstrated antagonistic effects, with a marked increase in LT_50_ (+2.96 days), indicating reduced efficacy under combined treatment (**Table 1**).

**Table 1:** Median lethal time (LT_50_) of entomopathogenic fungi applied alone or combined with plasma-activated water (PAW) against aphids.

Fungal species	Strain	ΔLT_50_ (UCL-LCL)		ΔLT_50_ (days)*
		EPF alone (days)	EPF + PAW (days)	
*Beauveria bassiana*	BbIPLCMU-1	7.83 (7.38 – 8.32)	10.79 (10.20 – 11.58)	+2.96
	BbIPLCMU-2	10.12 (9.69 – 10.64)	9.95 (9.32 – 10.75)	−0.17
	BbIPLCMU-18	7.31 (7.01 – 7.63)	7.14 (6.77 – 7.51)	−0.17
	SLLC-Bb 12	8.90 (8.45 – 9.41)	6.99 (6.59–7.39)	−1.91
*Cordyceps javanica*	SLLC-Cj 1	8.26 (7.64 – 8.90)	7.49 (7.20 – 7.78)	−0.77
	SLLC-Cj 2	7.89 (7.46 – 8.36)	8.69 (8.31 – 9.08)	+0.80
	SLLC-Cj 11	7.17 (6.90 – 7.44)	6.65 (6.02 – 7.27)	−0.52
	SLLC-Cj 24	6.93 (6.62 – 7.25)	6.60 (6.06–7.13)	−0.33
*Metarhizium anisopliae*	MaIPLCMU-5	/	10.37 (9.83 – 11.06)	/
	MaIPLCMU-10	/	8.83 (8.36 – 9.34)	/

LT_50_ values were estimated using probit regression analysis. The values are at 95% confidence intervals. As no mortality was observed with PAW alone, no data are presented here.

*ΔLT_50_ = Difference between combined treatment and EPF alone. Positive values (+) indicate delayed mortality under combined treatment, while negative value (−) indicates faster mortality under combined treatment. UCL and LCL refer to the upper and lower confidence limits of the estimated LT_50_, respectively./: represents the absence of values, due to not reaching the LT_50_ values during the experiment period.

## 4 Discussion

### 4.1 Characteristics of PAW and its repeated application on aphids

Aligning with previous studies (Xu et al., 2026; Zhao et al., 2026), this study also revealed strong insecticidal activity of PAW when applied directly to the tested mustard aphid. The strong acidification observed in this study (1.34–1.98 pH) likely played a central role in aphid mortality, as such low pH can disrupt cuticular integrity by influencing the activity of cuticle−associated enzymes (e.g., affecting proteases and hydrophobin expression) (St. Leger et al., 1998), impair cellular homeostasis (Harrison, 2001), and enhance oxidative stress (Messi et al., 2025) in insects.

Insect mortality also depends on physicochemical characteristics of the source water, concentration of PAW, and exposure time. Variations in water type (e.g., tap water, distilled water, or deionized water) can result in differences in conductivity, ion composition, and buffering capacity, thereby affecting RONS generation and stability, and ultimately the biological effectiveness of PAW. Nevertheless, deionized water has been reported to provide superior efficiency due to its low ionic background and minimal scavenging effects (Siddique et al., 2021a). However, the use of tap water in the present study also demonstrated sustained efficiency in generating reactive species in PAW, indicating its practical applicability under more realistic conditions.

Significant differences in PAW concentrations emerged by the third day, with higher concentrations associated with greater mortality. On the one hand, repeated applications play a key role in insect killing, particularly through sustained oxidative stress, which leads to a progressive increase in mortality (Ten Bosch et al., 2017). On the fifth day, cumulative mortality increased sharply across all PAW treatments, and on the seventh day, all insects had died regardless of concentration, supporting the aforesaid stress-related findings. Another notable feature of aphids is their thin cuticle and soft body, which may facilitate rapid penetration and internal accumulation of reactive species, leading to rapid mortality and potentially overcoming the defenses of hard-bodied insects (Wieczorek et al., 2019; Guo et al., 2025; Xu et al., 2026). In addition, PAW appeared to exhibit no neurotoxic effects in this study, as no sudden mortality or paralytic symptoms were observed during the treatment period.

Another notable feature of PAW is its safety for plants. Previous studies have shown that foliar applications of PAW do not cause damage to plant leaves despite their strongly acidic and oxidative properties (Kuzin et al., 2024). In contrast, PAW application has been reported to enhance fruit set, yield, and leaf nutrient content, particularly nitrogen and potassium, thereby promoting plant growth and development (Ramazzina et al., 2016; Kuzin et al., 2023). In the present study, the physicochemical changes associated with PAW application on plants were not particularly investigated. However, no visible signs of physical stress, such as discoloration, burning, or leaf shrinkage, were observed during the experimental period.

### 4.2 Effect of PAW on fungal conidial germination

This study demonstrates that fungal conidial germination is strongly regulated by the combined effects of PAW concentration and fungal strain, with significant interactions among those factors. At the lowest concentration (50 ppm), germination remained largely stable over time for most isolates, suggesting lower inhibitory activity under mild oxidative stress. In contrast, increasing concentrations (100 and 1,000 ppm) resulted in reductions in germination, particularly in susceptible isolates, confirming a clear dose- and exposure-dependent response. Importantly, the results indicate that germination responses are also slightly influenced by the prolonged exposure period (inhibiting effects in a strain-specific manner); however, not significant.

Similar findings have been reported previously; e.g., Siddique et al. (2021a) observed a significant reduction in germination of *Colletotrichum alienum* conidia under a 100 ppm PAW treatment. The inhibitory effect is likely associated with structural and ultrastructural damage, including cell wall maceration, cytoplasmic disorganization, vacuolar disruption, and nuclear and mitochondrial distortion (Siddique et al., 2021b). Furthermore, in a study, Los et al. (2020) demonstrated that PAW can disrupt fungal biofilm structures and inactivate spores of *Aspergillus flavus*, indicating broader antifungal activity. The destruction of conidia is particularly significant because conidial integrity is closely linked to subsequent hyphal and mycelial development; therefore, conidial damage may also reflect impaired mycelial growth potential (Ju et al., 2023).

### 4.3 Impact of the combined application of EPF and PAW on the mortality of the aphids

As previously described, except for a few strains, we didn’t observe a significant negative effect of PAW on conidial germination. As mentioned above, at 50 ppm, all isolates showed maximum conidial growth, though relatively strain-specific germination reduction was observed at 1,000 ppm of PAW. Thus, together with previous literature (Ki et al., 2020; Janda et al., 2021; Xu et al., 2026), we have tested the combined application of EPF with PAW at a moderate concentration of 100 ppm. Our findings suggest that moderate PAW concentrations are compatible with fungal propagules and may enable the simultaneous application of both agents without substantially compromising fungal infectivity. This compatibility is a key prerequisite for the development of integrated microbial–physical pest control strategies.

The concept of integrating EPF with complementary agents has been widely explored to improve the performance of biological control systems. Previous studies have demonstrated synergistic interactions between EPF and botanical insecticides, sublethal doses of chemical pesticides, and microbial metabolites (Fernández-Grandon et al., 2020; Alkhaibari et al., 2018). However, the integration of EPF with plasma-derived technologies remains largely unexplored. Even though the aforesaid complex mixture of reactive species present in PAW is increasingly recognized for its antimicrobial and insecticidal potential, yet their interactions with beneficial microorganisms such as EPF remain poorly understood (Laroussi, 2020; Wong et al., 2023). Therefore, the present study provides novel insights into the compatibility and potential synergistic interactions between EPF and PAW in insect pest management.

The standalone application of EPF resulted in high aphid mortality, with pronounced isolate-dependent variability. Such variation in virulence among fungal isolates is widely documented and is often associated with differences in host adhesion, cuticle penetration efficiency, extracellular enzyme production, and secondary metabolite synthesis (Vega et al., 2012; Parker et al., 2023; Rana et al., 2024). The variability in the infection-related traits of EPF can result in significant differences in pathogenicity even among isolates belonging to the same species (Zimmermann, 2007). In agreement with these observations, the present study identified highly virulent isolates such as *C. javanica* SLLC-Cj 24, which exhibited rapid aphid mortality under EPF-only treatment, whereas *M. anisopliae* MaIPLCMU-5 showed comparatively low pathogenicity.

When EPF was combined with PAW, aphid mortality generally increased and became more uniform across isolates, suggesting a potential enhancing or synergistic interaction between the two treatments. However, as no physiological or biochemical measurements were performed in the present study, the underlying mechanisms remain unclear. One possible explanation is that the reactive species present in PAW may compromise the structural integrity of the insect cuticle or induce physiological stress responses that increase host susceptibility to fungal infection. Reactive oxygen and nitrogen species are known to cause oxidative damage to lipids, proteins, and nucleic acids, thereby disrupting cellular homeostasis and weakening insect defense mechanisms (Guo et al., 2025). Therefore, these impacts could impair insect defense mechanisms. Such oxidative stress may also impair the insect immune system, including hemocyte-mediated phagocytosis and antimicrobial peptide production, which represent key components of the innate immune defense against fungal pathogens (Butt et al., 2016; Ortiz-Urquiza and Keyhani, 2016). When considered collectively, these results imply that exposure to PAW may induce physiological changes in the insect body, thereby promoting fungal colonization and growth. Nevertheless, additional experimental confirmation is necessary to support this argument.

Another possible mechanism involves PAW induced modification of the insect cuticle due to its acidic nature. The acidity of PAW could degrade the insect’s cuticular lipids and proteins, along with reactive radicals that normally act as protective barriers against microbial invasion. Such structural alterations could enhance fungal adhesion and penetration efficiency. Oxidative treatments can also increase cuticle permeability in insects, thereby promoting the entry of pathogens and toxins (González-Tokman et al., 2025). Thus, PAW may indirectly promote fungal infection by weakening the insect host’s physical and biochemical defenses.

Among the tested isolates, several strains appeared to benefit particularly from the combined treatment. For example, *M. anisopliae* MaIPLCMU-10 exhibited only moderate mortality under a single application but achieved complete mortality when combined with PAW. These findings suggest that PAW may enhance fungal virulence in certain isolates by facilitating early infection processes such as spore adhesion, germination on the host cuticle, or suppression of host immune responses. The enhancement of fungal pathogenicity under oxidative stress conditions has been reported in other host–pathogen systems, where stressed insects exhibit reduced immune competence and increased susceptibility to microbial infection (Braga et al., 2015).

Despite increased cumulative mortality observed across many isolates, the LT_50_ analysis showed that the combined treatment did not consistently accelerate insect mortality. In several isolates, PAW slightly increased LT_50_ values relative to EPF alone. This observation suggests that although PAW ultimately enhances cumulative mortality, it may transiently affect the early stages of fungal infection. Reactive oxygen species present in PAW could temporarily reduce conidial viability or delay germination on the insect surface, thereby slowing the initial establishment of fungal infection. Similar inhibitory effects of oxidative stress on fungal spore germination have been documented in several filamentous fungi (Zhang et al., 2020). Nevertheless, once infection was established, mortality reached high levels across most treatments, indicating that the overall biological impact of PAW remained beneficial in the combined system.

Interestingly, isolate *C. javanica* SLLC-Cj 2 exhibited reduced initial mortality under the combined treatment compared with EPF alone. This isolate-specific response highlights the complexity of interactions between plasma-derived reactive species and fungal pathogens. Differences in oxidative stress tolerance among fungal strains may influence their ability to survive and infect hosts in oxidative environments. Variations in antioxidant defense systems, including catalase, superoxide dismutase, and glutathione-based detoxification pathways, could determine the resilience of fungal propagules to PAW-induced oxidative stress (Braga et al., 2015). Therefore, the compatibility between EPF and PAW is also likely to be strain-specific, emphasizing the importance of screening fungal isolates before implementing integrated applications.

From an applied perspective, integrating PAW with EPF offers several potential advantages for sustainable pest management. First, PAW can directly suppress pest populations through oxidative stress while simultaneously enhancing the reliability of fungal biocontrol agents. Second, PAW is produced from water and atmospheric gases without synthetic chemicals, making it environmentally friendly and suitable for organic or low-input agricultural systems. Third, it has also shown greater cost-effectiveness in making PAW (**Supplementary Table 3**). Fourth, the combined approach may reduce isolate-dependent variability in fungal performance, thereby improving the consistency of biological control outcomes under field conditions. These characteristics make the EPF–PAW combination an attractive candidate for inclusion in integrated pest management (IPM) programs.

## 5 Limitations and future research directions

Although this study is limited to the laboratory, the results are promising; for future studies, it could also be tested in the field or greenhouse level. Based on the results, it is conceivable that new fungal strains are necessary for testing with PAW in combined applications, as the evidence shows strain-specific efficacy in combined treatments. Based on the repeated and single-time applications of PAW, it is clear that continuous application is needed for effective insect control. Therefore, future research should explore repeated PAW applications, with and without EPF, to further assess their potential for rapid insect mortality (concerning individual and combined applications). The efficacy of EPF–PAW combinations remains unclear, as most existing studies focus on single applications of either EPF (Bava et al., 2022; Ombura et al., 2025; Thanh et al., 2025) or PAW (Zhao et al., 2026) for insect control. This gap should be addressed in future research. Experiments should systematically compare combined EPF–PAW treatments with single treatments, particularly to identify potential synergistic or antagonistic effects. Future investigations should prioritize mechanistic studies of EPF–PAW–host insect interactions, along with gene-level understanding (Ahmad et al., 2025). Additionally, optimizing plasma generation parameters in laboratory studies may enable the production of PAW with customized chemical profiles that enhance insecticidal efficacy while minimizing damage to EPF. Moreover, future studies should also simulate field-level factors such as UV exposure and temperature regimes under controlled laboratory conditions to evaluate the efficacy of EPE, PAW, and the combination of both for a better understanding of their potential performance under real field conditions (Wu et al., 2020; Dias et al., 2021; Wu et al., 2023; Mehrabifard and Machala, 2025).

## 6 Conclusion

This study presents comprehensive evidence of the efficacy of naturally isolated EPF against aphids, a significant pest of economically important crops, under laboratory conditions. Plasma-activated water has emerged as a promising approach for managing these insect pests. Plasma treatment with a surface sDBD system produced chemically activated water enriched with stable reactive oxygen and nitrogen species, resulting in pronounced acidification and increased oxidative potential. Conidial germination assays demonstrated that low to moderate PAW concentrations (≤100 ppm) have little or no inhibitory effect on most isolates, supporting the compatibility of PAW with fungal biocontrol agents under optimized conditions. The PAW treatment with repeated application resulted in complete aphid mortality within seven days, most likely due to acidity, cumulative oxidative stress, rather than acute neurotoxicity. The entomopathogenic fungal isolates displayed considerable variability in virulence, as reflected by differences in cumulative mortality and LT_50_ values. The combined application of EPF and PAW significantly increased overall mortality in certain fungal strains and reduced variability among isolates, indicating a synergistic interaction (e.g., *B. bassiana* SLLC-Bb 12, *C. javanica* SLLC-Cj 1), although it did not significantly accelerate the rate of insect mortality. In certain strains, delayed LT_50_ values indicate that moderate oxidative exposure may temporarily affect early fungal establishment or conidial performance. From an applied perspective, PAW shows strong potential to improve the reliability and consistency of EPF-based pest management systems. However, synergistic effects are strain- and concentration-dependent and require precise calibration of concentration and exposure duration to maximize efficacy without adversely affecting infection dynamics.

## Data Availability

The original contributions presented in the study are included in the article/**Supplementary Material**. Further inquiries can be directed to the corresponding authors.
