# Supplementary material for: Entomopathogenic fungi, plasma-activated water, and their synergistic effects for sustainable management of Lipaphis erysimi
Source: Front Fungal Biol. 2026 Jun 30;7:1834224. doi: 10.3389/ffunb.2026.1834224 (PMC13365856; doi:10.3389/ffunb.2026.1834224)
Supplement: Supplementary file 1 [file Table1.docx]

Supplementary Material

**Supplementary Table 1.** Origin, host association, and isolation methods of entomopathogenic fungal isolates used in this study

| **Fungal species** | **Strain** | **Host** | **Host location** | **Isolation/collection method** | **Site of the collection** |
| --- | --- | --- | --- | --- | --- |
| *Beauveria bassiana* | BbIPLCMU-1 | Adult Aphid  (Hemiptera) | On leaf | Direct isolation from the infected host | Vegetable fields at Saraphi District, Chiang Mai Province, northern Thailand. |
|  | BbIPLCMU-2 | Adult Cicada  (Hemiptera) | On the plant stem |  | Khun Chang Khian Highland Research Station, Chiang Mai Province, northern Thailand |
|  | BbIPLCMU-18 | - | Loam soil (Rhizosphere soil, coffee plantation) | Insect baiting method using mealworm (*Tenebrio molitor*) larvae, followed by direct isolation |  |
|  | SLLC-Bb 12 | Adult beetle (Coleoptera) | On litter | Direct isolation from the infected host | Near association with Doi Inthanon National Park Mountain Forest, Chiang Mai Province, northern Thailand |
| *Cordyceps javanica* | SLLC-Cj 1 | Adult beetle  (Coleoptera) |  |  |  |
|  | SLLC-Cj 2 | Pupae  (Colecoptera) | In soil |  |  |
|  | SLLC-Cj 11 | Adult beetle  (Coleoptera) | On litter |  |  |
|  | SLLC-Cj 24 | Adult beetle (Coleoptera) | On litter |  |  |
| *Metarhizium anisopliae* | MaIPLCMU-5 | Beetle larvae (Coleoptera) | On the soil, potato cultivation land |  | Vegetable fields at Mae Wang District, Chiang Mai Province, northern Thailand |
|  | MaIPLCMU-10 | - | Sandy loam soil | Insect baiting method by using mealworm (*Tenebrio molitor*) larvae, followed by direct isolation |  |

Note: Pure cultures of the fungal isolates were obtained using either the single-spore or mycelial isolation method.

**Supplementary Table 2**. Operating parameters and experimental conditions used for the preparation of plasma-activated water using a surface dielectric barrier discharge (sDBD) plasma system.

| **Input Parameter** | **Operating condition/specification** |
| --- | --- |
| Water type | Tap water |
| Volume | 1,000 mL/batch |
| Plasma system | Surface dielectric barrier discharge (sDBD) |
| Working gas | Ambient air (no inlet flow) line |
| Power supply | Alternating current (AC), 220 V input |
| Output voltage | 3.5–5.0 kV (2 × 2.5 kV transformers) |
| Frequency | 50 Hz |
| Electric power | 220 W total (2 × 110 W transformer) |
| Electrode–water distance | 8 cm |
| Treatment time | 150 minutes |

**Supplementary Table 3**. Estimated electricity consumption (kWh) and operational electricity cost of the nitrogen radical generator based on sDBD electrodes.

| **Time** | **Electricity consumption (kWh)** | **Electricity cost** | |
| --- | --- | --- | --- |
|  |  | THB | USD |
| Hour | 0.22 | 0.88 | 0.027 |
| Day | 0.66 | 2.64 | 0.080 |
| Month | 19.8 | 79.2 | 2.410 |
| Year | 240.9 | 936.6 | 28.56 |

*Note: kWh: kilowatt-hour; THB: Thai Baht; USD: United States Dollar; Exchange rate: 1 USD= approx. 33 THB. Estimates were based on the production of 1 L of PAW at a concentration of 1,800–2,000 ppm after an hour of treatment.*
